# Hybrid Deep Learning–Machine Learning Fusion of Clinical, Radiomic and Deep Learning Features for Preoperative Differentiation of Solitary Pulmonary Mucinous Adenocarcinoma

**DOI:** 10.3390/diagnostics16111651

**Published:** 2026-05-27

**Authors:** Chao Sun, Jie Sun, Feng Wei, Shujie Yang, Weili Ba, Yiming Li

**Affiliations:** 1 Department of Radiology, Tianjin Union Medical Center, The First Affiliated Hospital of Nankai University, Tianjin 300121, China; sunchao_666@yeah.net (C.S.); 15201278157@163.com (F.W.); ysjie1022@163.com (S.Y.); 13622050698@163.com (W.B.); 2 Department of Pathology, Tianjin Union Medical Center, The First Affiliated Hospital of Nankai University, Tianjin 300121, China; sjdwgyx@126.com

**Keywords:** solitary pulmonary mucinous adenocarcinoma, deep learning, radiomics

## Abstract

**Objectives:** To develop and validate a hybrid deep learning–machine learning (DL-ML) fusion model for noninvasive preoperative differentiation of solitary pulmonary mucinous adenocarcinoma (SPMA). **Methods:** A total of 200 patients with pathologically confirmed lung adenocarcinoma, including 37 SPMA cases, treated at Tianjin Union Medical Center between 2018 and 2025 were retrospectively enrolled. Patients were randomly assigned to a training cohort (*n* = 140) and a test cohort (*n* = 60). Clinical characteristics, radiomic features, and deep learning features extracted via ResNet50 were integrated. Least absolute shrinkage and selection operator (LASSO) regression was used for feature selection, and multiple machine learning classifiers were compared. Model performance was assessed using the receiver operating characteristic (ROC) curve, decision curve analysis (DCA), and calibration curve analysis (CCA). **Results:** LASSO regression identified 10 optimal features, comprising 8 radiomic and 2 deep learning features. The random forest classifier yielded the best performance. The hybrid DL-ML fusion model yielded the highest AUC of 0.982 in the training cohort and 0.878 in the test cohort, significantly outperforming the clinical model (Clinic), radiomic model (Rad), deep transfer learning (DTL) model, deep learning–radiomics (DLR) model. In the test cohort, the hybrid DL-ML fusion model achieved an AUC of 0.878, which was significantly higher than that of the clinical model (0.755; *p* < 0.05). DCA and CCA confirmed favorable clinical utility and calibration. **Conclusions:** The hybrid DL-ML fusion model enables accurate, noninvasive preoperative differentiation of SPMA. It outperforms conventional clinical assessment and single-modality imaging models, with promising potential for noninvasive preoperative differential diagnosis.

## 1. Introduction

Lung cancer is the leading cause of cancer-related mortality globally, with lung adenocarcinoma being the most common histological subtype. Pulmonary mucinous adenocarcinoma (PMA) is a rare but biologically distinct variant, accounting for 0.2–5% of all lung adenocarcinomas [1,2,3]. It is characterized by abundant extracellular mucin secretion, mucin lake formation, and low cellular atypia, with columnar or goblet cell morphology [4,5,6]. Despite its relatively indolent histopathological appearance, PMA is highly invasive, prone to pleural invasion, lymph node metastasis, and spread through air spaces, resulting in poor prognosis and heterogeneous treatment responses [7,8,9,10]. As a special subtype of PMA, SPMA has an insidious onset and atypical clinical and imaging manifestations, which easily cause delayed diagnosis in clinical practice [11,12].

Chest CT is the first-line imaging modality for evaluating solitary pulmonary nodules. However, CT features of SPMA overlap extensively with benign lesions such as tuberculoma and inflammatory pseudotumor, as well as non-mucinous adenocarcinoma [13,14]. SPMA typically manifests as solid or subsolid nodules with homogeneous density, lacking pathognomonic imaging signs [15,16]. Consequently, the preoperative misdiagnosis rate remains high, contributing to inappropriate clinical management and suboptimal patient outcomes.

While CT-guided biopsy and surgical resection enable definitive pathological confirmation, these invasive procedures carry risks of bleeding, infection, and tumor seeding. They are also unsuitable for widespread preoperative screening or risk stratification of indeterminate SPMA. In the era of precision oncology, radiomics and deep learning have emerged as powerful tools for noninvasive tumor characterization [17,18]. These approaches extract high-throughput quantitative imaging features that reflect the underlying histopathological and molecular heterogeneity of tumors [19,20,21]. Recent studies have demonstrated the utility of CT-based radiomics in differentiating SPMA from solitary pulmonary nonmucinous adenocarcinoma (SPNMA) or benign pulmonary lesions [22,23,24,25]. Despite these advances, critical gaps persist: existing models rely primarily on radiomic features or limited clinical parameters, lacking a systematic hybrid fusion framework. Furthermore, few studies have rigorously compared multiple machine learning classifiers to identify the optimal algorithm for SPMA detection.

Accordingly, this study aimed to develop and validate a noninvasive, robust hybrid DL-ML fusion model that integrates clinical characteristics, CT radiomic features, and deep learning features for accurate preoperative differentiation of SPMA. The proposed model may facilitate early and precise diagnosis in patients with SPMA.

## 2. Methods

### 2.1. Study Population

This retrospective observational study was conducted in accordance with the Declaration of Helsinki. Patients with pathologically confirmed lung adenocarcinoma treated at Tianjin Union Medical Center (The First Affiliated Hospital of Nankai University) between January 2018 and December 2025 were retrospectively reviewed. A total of 200 patients were included. Inclusion criteria were: (1) histopathological diagnosis of lung adenocarcinoma; (2) availability of complete chest CT imaging. Exclusion criteria were: (1) lesion diameter < 1.0 cm; (2) images with severe artifacts; (3) history of chemotherapy or radiotherapy prior to CT examination or pathological diagnosis.

### 2.2. CT Image Acquisition

All patients underwent non-contrast chest CT using multi-detector scanners (Optima CT660 64-slice, GE Healthcare, Milwaukee, WI, USA; Aquilion 64-slice, Canon Medical Systems, Otawara, Japan; or Aquilion ONE 320-slice, Canon Medical Systems, Otawara, Japan). Scanning parameters were as follows: tube voltage, 120 kV; tube current, 140 mAs; scan range, from the thoracic inlet to the lung base; slice thickness and interval, 5 mm; pitch, 1.0; 1-mm thin-slice reconstruction. Images were acquired during inspiratory breath-holding in the supine position. Only non-contrast CT images were analyzed; contrast-enhanced CT examinations were excluded.

### 2.3. Image Processing and Segmentation

To ensure analytical consistency, all CT images were normalized to reduce inter-scanner variability and resampled to an isotropic voxel size of 1 mm × 1 mm × 1 mm. Region-of-interest (ROI) segmentation was performed using ITK-SNAP software (version 3.8.0; http://www.itksnap.org). Lung window settings were used to optimize visualization of tumor boundaries. After initial delineation by one radiologist, all segmentations were reviewed by a second radiologist, with consensus required before exporting the maximum cross-sectional tumor image. Feature reproducibility was evaluated using the intraclass correlation coefficient (ICC); an ICC > 0.85 was considered to indicate excellent agreement.

### 2.4. Feature Extraction and Model Construction

**Figure 1:**
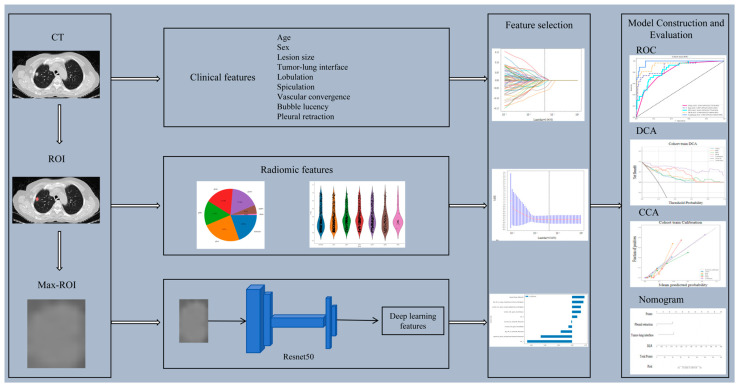
Study workflow. First, CT images were preprocessed, including ROI segmentation and Max-ROI cropping. Next, three types of features were extracted: clinical features, radiomic features, and deep learning features obtained via the ResNet50 network. Then, feature selection was performed to identify the most informative features. Finally, prediction models were constructed and evaluated using ROC curves, DCA, CCA, and nomograms. ROI, region of interest; Max-ROI, maximum cross-sectional region of interest; ResNet50, deep residual network; DCA, Decision curve analysis; CCA, Calibration curve analysis.

Five categories of features were extracted: clinical characteristics, radiomic features, deep learning features, deep learning–radiomics fusion features, and combined fusion features (Figure 1). Multivariable logistic regression was used for clinical feature selection. A total of 1834 radiomic features were extracted using the PyRadiomics package (version 3.0.1; https://pyradiomics.readthedocs.io (accessed on 20 February 2026)). A total of 2049 deep learning features were derived using ResNet50 (https://pytorch.org/hub/pytorch_vision_resnet (accessed on 23 February 2026), which was employed solely for feature extraction and not for model training or classification. All features were normalized using Z-score standardization. Given the rarity of SPMA and the relatively small sample size, multiple strategies were implemented to mitigate overfitting: (1) 10-fold cross-validation was applied throughout model development for feature screening, hyperparameter tuning, and model optimization; no independent fixed validation set was separated to maximize data utilization. (2) Prior to model training, Pearson correlation analysis was performed to remove highly redundant features with correlation coefficients > 0.9, achieving preliminary dimensionality reduction and alleviating multicollinearity. (3) Further feature selection and overfitting control were performed via LASSO regression with L1 regularization, which penalizes feature coefficients and drives trivial coefficients to zero for automatic feature simplification. The optimal λ value was determined via 10-fold cross-validation to balance model complexity and generalization. Seven machine learning classifiers were evaluated: logistic regression (LR), support vector machine (SVM), K-nearest neighbors (KNN), random forest, extremely randomized trees (ExtraTrees), light gradient boosting machine (LightGBM), and multi-layer perceptron (MLP). The optimal classifier was selected to construct five diagnostic models: Clinic, Rad, DTL, DLR, and hybrid DL-ML fusion model.

### 2.5. Model Evaluation and Nomogram Construction

The optimal hybrid DL-ML model was evaluated using ROC curve analysis, AUC, DCA, and CCA. A nomogram was developed based on the optimal model.

### 2.6. Statistical Analysis

Statistical analyses were performed using SPSS software (version 21.0; IBM Corp., Armonk, NY, USA). Normally distributed continuous variables are expressed as mean ± standard deviation and compared using the independent samples *t*-test; non-normally distributed continuous variables are presented as median (interquartile range) and analyzed using the Mann–Whitney U test. Categorical variables were compared using the χ^2^ test or Fisher’s exact test. Pairwise comparisons of AUC values between models were performed using the DeLong test. A two-sided *p* < 0.05 was considered statistically significant. For model training and feature selection, 10-fold cross-validation was applied in the training cohort to mitigate overfitting and optimize parameters; the held-out test cohort was used for independent validation.

## 3. Results

### 3.1. Patient Characteristics

A total of 200 patients with pathologically confirmed lung adenocarcinoma were enrolled in this study, comprising 91 males (45.5%) and 109 females (54.5%), with a mean age of 66.60 ± 9.09 years. All patients were of Chinese Han ethnicity. The cohort included 37 cases (18.5%) of SPMA and 163 cases (81.5%) of SPNMA. Given the class imbalance, stratified random sampling was applied to preserve a similar subtype proportion in the training and test cohorts. Patients were randomly allocated at a 7:3 ratio into a training cohort (140 patients) and a test cohort (60 patients). The baseline demographic and clinical characteristics were well-balanced between the training and test cohorts (Table 1). No significant differences were observed in age, sex distribution, or lesion size between the two cohorts, confirming successful randomization and adequate group comparability.

Comparisons between the SPMA and SPNMA groups revealed significant differences in two key imaging features. SPMA demonstrated a significantly higher frequency of indistinct tumor-lung interface compared with SPNMA. Conversely, pleural retraction was significantly less common in SPMA than in SPNMA. Other imaging characteristics, including lesion size, lobulation, spiculation, vascular convergence, and bubble lucency, showed no statistically significant differences between the two histological subtypes (all *p* > 0.05).

### 3.2. Clinical Feature Analysis and Model Construction

Multivariable logistic regression analysis identified two independent clinical predictors of SPMA (Table 2). An indistinct tumor-lung interface emerged as a significant protective factor (odds ratio [OR], 0.220; 95% confidence interval [CI], 0.091–0.532; *p* = 0.005). Pleural retraction demonstrated the strongest protective association (OR, 0.147; 95% CI, 0.064–0.341; *p* < 0.001). Neither demographic variables nor other imaging features showed independent predictive value in the multivariable model. The clinical prediction model incorporating these two independent predictors achieved an AUC of 0.814 (95% CI, 0.742–0.886) in the training cohort and 0.755 (95% CI, 0.623–0.887) in the test cohort.

### 3.3. Radiomic and Deep Learning Feature Extraction, Feature Selection and Dimensionality Reduction

**Figure 2:**
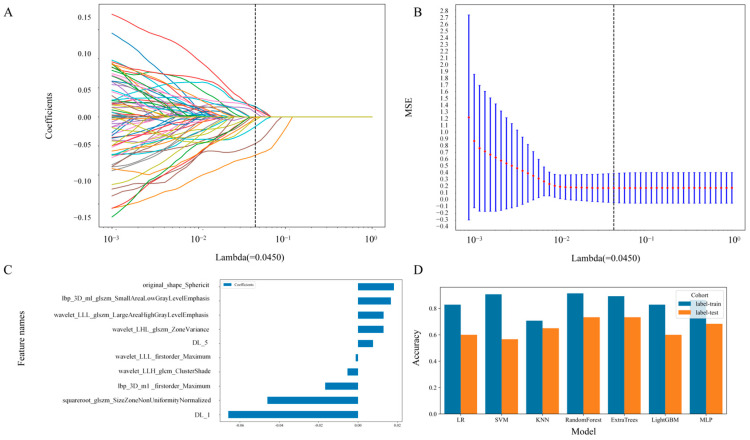
Deep learning-radiomics feature extraction and diagnostic performance of different machine learning models for solitary SPMA. (**A**) Coefficient path plot; (**B**) Cross-validation curve; (**C**) Histogram of optimal feature coefficients; (**D**) Accuracy of different machine learning models.

High-throughput feature extraction using PyRadiomics (version 3.0.1) yielded 1834 radiomic features. Deep feature extraction was performed using ResNet50 pre-trained on ImageNet, generating 2049 features from the final fully connected layer. Feature fusion was then conducted to combine these radiomic and deep learning features, resulting in a total of 3883 features. LASSO regression with 10-fold cross-validation was applied to the combined feature set, resulting in a parsimonious set of 10 features with non-zero coefficients (Figure 2A–C). The finally selected features included 2 deep learning features and 8 radiomic features, which were derived from shape features, wavelet transforms, local binary pattern texture features, and square-root filtering.

### 3.4. Classifier Selection and Model Optimization

In this study, we systematically evaluated the classification performance of seven mainstream machine learning models on a small-sample clinical dataset (Figure 2D). Given the imbalanced nature of small-sample clinical data, the Precision-Recall Area Under the Curve (PR-AUC) was selected as the primary evaluation metric to avoid overestimation of model performance that may be caused by the ROC-AUC. Accuracy, F1-score, precision, and recall were used as supplementary evaluation metrics (Table 3). The test set results showed that the Random Forest model comprehensively outperformed all other comparative models across all core evaluation metrics: it achieved the highest test set PR-AUC of 0.7510 (6.85 percentage points higher than the second-ranked MLP model), the highest ROC-AUC of 0.824, the highest F1-score of 0.7774, the highest precision of 0.7834, the highest recall of 0.7715, and an accuracy of 0.7333 (tied for the highest among all models). Further analysis of model generalization ability revealed that the Random Forest model exhibited the lowest degree of overfitting, with only a 10-percentage-point decrease in PR-AUC from the training set to the test set. In sharp contrast, models such as SVM, ExtraTrees, and LightGBM showed severe overfitting, with PR-AUC attenuation exceeding 17 percentage points. Based on the comprehensive evaluation of clinical practicality, generalization ability, and overfitting risk, the Random Forest model was finally determined as the optimal classifier for this small-sample clinical case classification task.

### 3.5. Comprehensive Model Performance Evaluation and Model Performance

**Figure 3:**
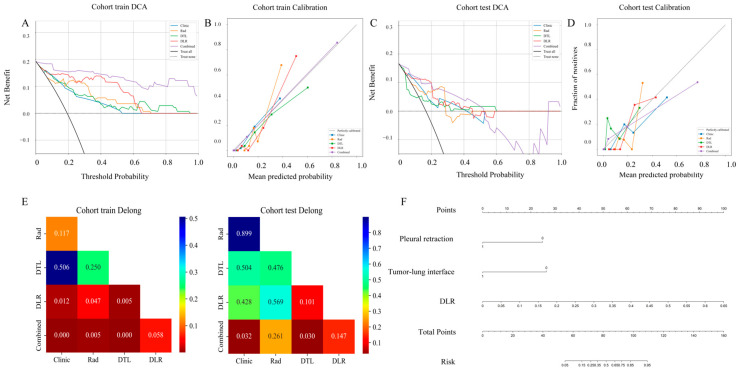
DCA, CCA, inter-model performance comparison, and nomogram construction for the prediction of SPMA. (**A**) DCA curves in the training cohort; (**B**) Calibration curves in the training cohort; (**C**) DCA curves in the test cohort; (**D**) Calibration curves in the test cohort; (**E**) Heatmaps of DeLong test results for pairwise inter-model AUC comparison in the training (left) and test (right) cohorts; (**F**) Nomogram constructed based on the optimal model for individualized solitary SPMA risk prediction. Clinic: clinical model; Rad: radiomics model; DTL: deep transfer learning model; DLR: deep learning-radiomics model; Combined: hybrid DL-ML fusion model.

Five distinct prediction models were constructed and comparatively evaluated, with the hybrid DL-ML fusion model showing the best performance. In the training cohort, the clinical, radiomics, DTL, DLR, and fusion models achieved AUCs of 0.814, 0.897, 0.851, 0.944, and 0.982, respectively. In the test cohort, the fusion model achieved an AUC of 0.878, outperforming the clinical model (AUC, 0.755), radiomics model (AUC, 0.771), DTL model (AUC, 0.678), and DLR model (AUC, 0.824). In the training cohort, the fusion model achieved a precision of 0.872, recall of 0.852, F1-score of 0.862, and PR-AUC of 0.927. In the test cohort, the fusion model achieved a precision of 0.783, recall of 0.761, F1-score of 0.772, and PR-AUC of 0.846, indicating reliable performance in the imbalanced clinical setting (Table 4). DCA was used to evaluate clinical utility across different threshold probabilities (Figure 3A,C). In both cohorts, the hybrid DL-ML fusion model demonstrated a superior net benefit compared with all comparator models. CCA revealed excellent agreement between the predicted probabilities and observed outcomes for the fusion model (Figure 3B,D). Quantitative calibration assessment was performed using the Brier score and calibration slope. In the training cohort, the hybrid DL-ML fusion model had a Brier score of 0.068 and a calibration slope of 0.924. In the test cohort, the Brier score was 0.132 and the calibration slope was 0.876, indicating excellent agreement between predicted and observed probabilities. The DeLong test in the test cohort demonstrated that the hybrid DL-ML fusion model significantly outperformed the clinical model (*p* = 0.042) and the DTL model (*p* = 0.018), with marginal superiority over the radiomics model (*p* = 0.076) and the DLR model (*p* = 0.189) (Figure 3E).

### 3.6. Nomogram Development

Based on the hybrid DL-ML fusion model, a clinically applicable nomogram was developed, which incorporated the two clinical predictors (indistinct tumor-lung interface and pleural retraction) and the 10 selected radiomic and deep learning features (Figure 3F). This nomogram enables individualized risk estimation of SPMA by summing the assigned points for each predictor variable.

## 4. Discussion

This study developed and validated a hybrid DL-ML fusion model integrating clinical characteristics, radiomic features, and deep learning representations for the preoperative differentiation of solitary SPMA from SPNMA. Our principal findings are as follows (1) an indistinct tumor-lung interface and the absence of pleural retraction are independent clinical predictors of SPMA; (2) random forest-optimized feature selection yielded a parsimonious set of 10 predictive variables (8 radiomic and 2 deep learning features); (3) the hybrid DL-ML fusion model achieved exceptional diagnostic performance, with AUCs of 0.982 and 0.878 in the training and test cohorts, respectively; (4) DCA confirmed a superior clinical net benefit of the fusion approach across clinically relevant threshold probabilities; and (5) the developed nomogram enables individualized risk stratification for clinical application. These results confirm that the hybrid DL-ML fusion approach significantly improves the diagnostic accuracy of SPMA compared with conventional clinical assessments or single-modality imaging evaluations.

Multivariable analysis identified two CT morphological features as independent predictors of SPMA, providing mechanistic insights into the pathophysiological basis of this distinct subtype. The strong association between an indistinct tumor-lung interface and SPMA is consistent with the characteristic histopathology of mucin-rich tumors. SPMA is characterized by abundant extracellular mucin production by neoplastic columnar and goblet cells, with frequent spillage into adjacent alveolar spaces. Mucin dissemination forms a transitional zone of tumor infiltration and inflammatory response at the lesion periphery, which manifests radiologically as ill-defined margins. In contrast, SPNMA typically exhibits a more cohesive growth pattern with sharper demarcation from the surrounding lung parenchyma [16].

The protective association of pleural retraction reflects fundamental differences in tumor-stromal interactions. Pleural retraction arises from fibroblastic proliferation and desmoplastic reactions within the tumor microenvironment, generating contractile forces that distort adjacent pleural surfaces [26,27]. The mucin-dominant composition of SPMA, characterized by relatively sparse cellular components and minimal stromal reaction, explains the infrequent occurrence of this sign [28]. Notably, indicators including spiculation, lobulation, and vascular convergence showed no independent predictive value in our cohort, which is inconsistent with the findings of Qi et al. [29]. It is well established that CT features correlate with clinical outcomes in patients with SPMA. Consistent with this, Lee et al. demonstrated that visual FDG avidity on ^18^F-FDG PET/CT, when combined with CT morphological phenotypes, improved prognostic stratification in patients with curatively resected PMA; both FDG-avid lesions and the pneumonic-type phenotype were associated with significantly worse disease-free survival [30]. Although our current study did not evaluate the prognostic impact of these imaging findings, future studies will aim to clarify their correlation with long-term clinical outcomes in this patient population.

The superior performance of the Random Forest algorithm stems from its ensemble learning design, which inherently mitigates overfitting. Bootstrap aggregation generates diverse training subsets, while random feature selection decorrelates individual trees. Averaging tree predictions reduces variance and the risk of overfitting, making it ideal for our small, high-dimensional dataset. The Random Forest algorithm mitigates overfitting through two core ensemble strategies: (1) bootstrap aggregation, which generates diverse training subsets by random sampling with replacement to reduce model variance; and (2) random feature selection, which splits nodes using a random subset of features to decorrelate individual trees. Averaging predictions across independent trees suppresses noise and enhances generalization [31,32]. Notably, the Random Forest algorithm achieved near-optimal results with default parameters, providing practical advantages for clinical translation [33,34,35].

Our findings advance previous investigations on the imaging characterization of SPMA. Xiao et al. reported a radiomics AUC of 0.829 for differentiating mucinous from non-mucinous adenocarcinoma in nodules ≤ 3 cm, which is comparable to the AUC of our radiomics model (test AUC, 0.771) [36]. However, their study excluded deep learning, limiting its integration into comprehensive assessments. Hong et al. developed a combined clinical-radiomic model with an AUC of 0.84 but lacked deep learning components [37]. Zhong et al. constructed a combined clinical-radiomic model with an AUC of 0.83, which was lower than that of the model established in the present study [38]. Zhang et al. demonstrated that a combined model integrating contrast-enhanced CT radiomic features and clinical parameters could accurately predict invasive SPMA, with AUCs of 0.840 (training) and 0.850 (testing), outperforming standalone clinical or radiomic models [27]. Notably, our study used unenhanced plain CT and achieved comparable predictive performance. Similarly, our integrated model also exhibited superior predictive advantages over single-factor models.

A careful comparison of training and test performance confirms the robustness and generalizability of the model. The high training AUC (0.982) reflects strong fitting ability on the labeled dataset, while the test AUC (0.878) demonstrates stable performance on unseen cases. The moderate AUC reduction (0.104) is typical in radiomic studies with limited sample sizes and indicates no severe overfitting. Importantly, the test-set AUC remained above 0.85 and outperformed all comparator models, confirming its reliable generalizability and clinical utility.

The incremental value of our fusion model is evidenced by the progressive improvement in AUC from 0.755 (clinical model) to 0.878 (hybrid DL-ML fusion model). This 0.123 AUC increase from the clinical model to the combined model in the test cohort represents a clinically meaningful improvement, especially considering the diagnostic challenge of SPMA. The test AUC of 0.878 suggests the potential of this model for noninvasive preoperative stratification.

Regarding sample size adequacy, our cohort is consistent with recently published radiomic studies focusing on rare lung adenocarcinoma subtypes. Previous SPMA-related imaging studies typically included 30–60 SPMA cases, as this subtype accounts for only 0.2–5% of all lung adenocarcinomas [23,36,38,39]. To address potential overfitting in the small and imbalanced SPMA cohort, we integrated multiple complementary strategies: 10-fold cross-validation ensured stable feature selection and hyperparameter optimization without data leakage; feature redundancy reduction (r > 0.9) and LASSO regularization minimized model complexity; and the selection of Random Forest, an ensemble classifier with built-in variance reduction, further mitigated overfitting. Validation with an independent test cohort confirmed that these strategies collectively enhanced the robustness and generalizability of the model.

This study has several limitations. First, its retrospective single-center design introduces selection bias and limits generalizability to other institutions with different CT protocols and patient populations. External validation using a completely independent multicenter cohort was not performed, which is the major limitation in evaluating the model’s generalization performance. Although our multi-scanner approach partially addresses hardware variability, standardized prospective image acquisition would enhance reproducibility. Second, the modest SPMA sample size (*n* = 37, 18.5%), which reflects the rarity of this subtype, constrains the stability of feature selection and the power of subgroup analyses. Third, this study only included non-contrast chest CT images. Most existing deep learning and radiomic studies on pulmonary nodule characterization also use non-contrast CT, due to its wide accessibility, routine application in lung cancer screening, and freedom from contrast-induced artifacts that compromise texture and radiomic feature quantification. However, the model cannot be directly extended to contrast-enhanced CT (e.g., portal venous phase scans), as iodine contrast alters tissue attenuation and reshapes the distribution of radiomic and deep learning features. Future studies will incorporate larger sample sizes, multicenter designs, prospective cohorts, contrast-enhanced CT datasets, and rigorous external validation to further verify the robustness and generalizability of the proposed fusion model.

## 5. Conclusions

We developed and validated a hybrid DL-ML radiomic fusion model that achieves highly accurate, noninvasive preoperative prediction of SPMA. By synergistically integrating clinical CT features, quantitative radiomic biomarkers, and deep learning representations, the model overcomes the limitations of individual assessment modalities alone and provides clinically actionable and reliable risk stratification. The proposed approach lays a solid foundation for the precise diagnosis of SPMA, which may further support individualized surgical planning and patient-specific tailored therapeutic strategies.

## Figures and Tables

**Table 1:** Patient Characteristics and Imaging Features of SPMA.

Characteristic	Training Cohort		*p*	Test Cohort		*p*
	SPNMA (*n* = 113)	SPMA (*n* = 27)		SPNMA (*n* = 50)	SPMA (*n* = 10)	
Age (years)	66.77 ± 9.27	65.15 ± 7.32	0.398	67.48 ± 9.39	64.20 ± 10.34	0.325
Sex, n (%)			0.909			0.729
Female	63 (55.75)	16 (59.26)		26 (52.00)	4 (40.00)	
Male	50 (44.25)	11 (40.74)		24 (48.00)	6 (60.00)	
Lesion size (cm)	1.86 ± 0.59	1.94 ± 0.67	0.521	1.80 ± 0.66	1.62 ± 0.57	0.421
Tumor-lung interface, *n* (%)			0.002			0.037
Indistinct	40 (35.40)	19 (70.37)		19 (38.00)	8 (80.00)	
Distinct	73 (64.60)	8 (29.63)		31 (62.00)	2 (20.00)	
Lobulation, *n* (%)			0.459			0.804
Absent	39 (34.51)	12 (44.44)		15 (30.00)	4 (40.00)	
Present	74 (65.49)	15 (55.56)		35 (70.00)	6 (60.00)	
Spiculation, *n* (%)			0.155			0.057
Absent	28 (24.78)	11 (40.74)		16 (32.00)	7 (70.00)	
Present	85 (75.22)	16 (59.26)		34 (68.00)	3 (30.00)	
Vascular convergence, *n* (%)			0.065			0.418
Absent	54 (47.79)	7 (25.93)		25 (50.00)	3 (30.00)	
Present	59 (52.21)	20 (74.07)		25 (50.00)	7 (70.00)	
Bubble lucency, *n* (%)			0.767			0.077
Absent	40 (35.40)	11 (40.74)		23 (46.00)	1 (10.00)	
Present	73 (64.60)	16 (59.26)		27 (54.00)	9 (90.00)	
Pleural retraction, *n* (%)			<0.001			0.164
Absent	27 (23.89)	19 (70.37)		20 (40.00)	7 (70.00)	
Present	86 (76.11)	8 (29.63)		30 (60.00)	3 (30.00)	

SPNMA, solitary pulmonary non-mucinous adenocarcinoma; SPMA, solitary pulmonary mucinous adenocarcinoma.

**Table 2:** Multivariable Logistic Regression Analysis for the Prediction of SPMA.

Characteristic	OR (95% CI)	*p*
Age (years)	0.996 (0.973–1.020)	0.793
Sex	0.980 (0.424–2.261)	0.968
Lesion size (cm)	1.367 (0.708–2.643)	0.435
Tumor-lung interface	0.220 (0.091–0.532)	0.005
Lobulation	0.874 (0.379–2.016)	0.791
Spiculation	0.630 (0.267–1.490)	0.378
Vascular convergence	2.295 (0.934–5.641)	0.129
Bubble lucency	0.744 (0.320–1.732)	0.565
Pleural retraction	0.147 (0.064–0.341)	<0.001

OR, odds ratio; CI, confidence interval.

**Table 3:** Diagnostic Performance of Different Machine Learning Models for SPMA.

Model	Cohort	Accuracy	AUC	95% CI	Precision	Recall	F1	PR-AUC
LR	Training	0.8286	0.8755	0.8067–0.9442	0.8281	0.8123	0.8201	0.7621
	Test	0.6	0.742	0.6104–0.8736	0.63	0.5832	0.6057	0.6210
SVM	Training	0.9071	0.9092	0.8325–0.9860	0.9922	0.8265	0.9018	0.7987
	Test	0.5667	0.682	0.5357–0.8283	0.5665	0.5521	0.5592	0.5605
KNN	Training	0.7071	0.8653	0.8079–0.9226	0.7073	0.6925	0.6998	0.7512
	Test	0.65	0.733	0.5900–0.8760	0.6509	0.6382	0.6445	0.6120
Random Forest	Training	0.9143	0.9436	0.8938–0.9934	0.9856	0.8698	0.9241	0.8385
	Test	0.7333	0.824	0.7139–0.9341	0.7834	0.7715	0.7774	0.7510
ExtraTrees	Training	0.8929	0.9218	0.8523–0.9913	0.9381	0.8412	0.8870	0.8105
	Test	0.7333	0.71	0.5840–0.8360	0.7334	0.7205	0.7269	0.5902
LightGBM	Training	0.8286	0.9308	0.8842–0.9774	0.8287	0.8156	0.8221	0.8203
	Test	0.6	0.77	0.6304–0.9096	0.6967	0.5165	0.5932	0.6511
MLP	Training	0.8643	0.8587	0.7754–0.9421	0.9228	0.8012	0.8577	0.7452
	Test	0.6833	0.804	0.6872–0.9208	0.6834	0.6705	0.6769	0.6825

LR, logistic regression; SVM, support vector machine; KNN, K-nearest neighbors; ExtraTrees, extremely randomized trees; LightGBM, light gradient boosting machine; MLP, multi-layer perceptron; AUC, area under the curve; CI, confidence interval.

**Table 4:** Diagnostic Performance of Different Models for SPMA.

Model	Cohort	Accuracy	AUC	95% CI	Precision	Recall	F1	PR-AUC
Clinic	Training	0.800	0.814	0.735–0.893	0.803	0.794	0.798	0.811
	Test	0.750	0.755	0.659–0.851	0.747	0.730	0.738	0.752
Rad	Training	0.886	0.897	0.829–0.965	0.880	0.871	0.875	0.812
	Test	0.754	0.771	0.617–0.925	0.763	0.750	0.756	0.708
DTL	Training	0.686	0.851	0.779–0.923	0.843	0.836	0.839	0.818
	Test	0.671	0.678	0.551–0.805	0.668	0.650	0.659	0.625
DLR	Training	0.914	0.944	0.894–0.993	0.939	0.925	0.932	0.919
	Test	0.733	0.824	0.714–0.934	0.815	0.804	0.809	0.801
Combined	Training	0.914	0.982	0.966–0.999	0.872	0.852	0.862	0.927
	Test	0.833	0.878	0.785–0.971	0.783	0.761	0.772	0.846

Clinic, clinical model; Rad, radiomics model; DTL, deep transfer learning model; DLR, deep learning-radiomics model; Combined, hybrid DL-ML fusion model.

## Data Availability

The raw/processed data and underlying code used in this study are not publicly available due to institutional policy, patient privacy restrictions, and ethical constraints. However, reasonable requests for data and code can be sent to the corresponding author for individual academic non-commercial research purposes. Access will be granted after formal application and institutional approval.

## Abbreviations

AUC	Area under the curve
CCA	Calibration curve analysis
CI	Confidence interval
DTL	Deep transfer learning
DLR	Deep learning-radiomics
DL-ML	Deep learning machine learning
DCA	Decision curve analysis
ExtraTrees	Extremely randomized trees
ICC	Intraclass correlation coefficient
KNN	K-nearest neighbors
LR	Logistic regression
LASSO	Least absolute shrinkage and selection operator
LightGBM	Light gradient boosting machine
MLP	Multi-layer perceptron
OR	Odds ratio
PMA	Pulmonary mucinous adenocarcinoma
ROI	Region-of-interest
ROC	Receiver operating characteristic
PR-AUC	Precision-Recall Area Under the Curve
SPMA	Solitary pulmonary mucinous adenocarcinoma
SPNMA	Solitary pulmonary non-mucinous adenocarcinoma
SVM	Support vector machine
